# Intra- and peri-tumoral MRI radiomics features for preoperative lymph node metastasis prediction in early-stage cervical cancer

**DOI:** 10.1186/s13244-023-01405-w

**Published:** 2023-04-15

**Authors:** Zhenhua Zhang, Xiaojie Wan, Xiyao Lei, Yibo Wu, Ji Zhang, Yao Ai, Bing Yu, Xinmiao Liu, Juebin Jin, Congying Xie, Xiance Jin

**Affiliations:** 1 Department of Radiology, The 1st Affiliated Hospital of Wenzhou Medical University, Wenzhou, China; 2 Department of Obstetrics and Gynecology, Women’s Hospital, Zhejiang University School of Medicine, Hangzhou, China; 3 Department of Radiotherapy Center, The 1st Affiliated Hospital of Wenzhou Medical University, Wenzhou, China; 4 School of Laboratory Medicine and Life Sciences, Wenzhou Medical University, Wenzhou, China; 5 Department of Medical Engineering, The 1st Affiliated Hospital of Wenzhou Medical University, Wenzhou, China; 6 Department of Radiation and Medical Oncology, The 2nd Affiliated Hospital of Wenzhou Medical University, Wenzhou, China; 7 School of Basic Medical Science, Wenzhou Medical University, Wenzhou, China

**Keywords:** Early-stage cervical cancer, Lymph nodes metastasis, Radiomics, Magnetic resonance imaging, Peritumoral region

## Abstract

**Background:**

Noninvasive and accurate prediction of lymph node metastasis (LNM) is very important for patients with early-stage cervical cancer (ECC). Our study aimed to investigate the accuracy and sensitivity of radiomics models with features extracted from both intra- and peritumoral regions in magnetic resonance imaging (MRI) with T2 weighted imaging (T2WI) and diffusion weighted imaging (DWI) for predicting LNM.

**Methods:**

A total of 247 ECC patients with confirmed lymph node status were enrolled retrospectively and randomly divided into training (*n* = 172) and testing sets (*n* = 75). Radiomics features were extracted from both intra- and peritumoral regions with different expansion dimensions (3, 5, and 7 mm) in T2WI and DWI. Radiomics signature and combined radiomics models were constructed with selected features. A nomogram was also constructed by combining radiomics model with clinical factors for predicting LNM.

**Results:**

The area under curves (AUCs) of radiomics signature with features from tumors in T2WI and DWI were 0.841 vs. 0.791 and 0.820 vs. 0.771 in the training and testing sets, respectively. Combining radiomics features from tumors in the T2WI, DWI and peritumoral 3 mm expansion in T2WI achieved the best performance with an AUC of 0.868 and 0.846 in the training and testing sets, respectively. A nomogram combining age and maximum tumor diameter (MTD) with radiomics signature achieved a C-index of 0.884 in the prediction of LNM for ECC.

**Conclusions:**

Radiomics features extracted from both intra- and peritumoral regions in T2WI and DWI are feasible and promising for the preoperative prediction of LNM for patients with ECC.

**Supplementary Information:**

The online version contains supplementary material available at 10.1186/s13244-023-01405-w.

## Key points

1. Radiomics models with features from MRI for LNM prediction for ECC.
2. Combined radiomics signature with intra- and peritumoral regions achieved the best performance.
3. Combined radiomics signature with an AUC of 0.846 for predicting LNM.
4. A nomogram combining clinical factors with radiomics signature further improves predictive performance.

**Supplementary Information:**

The online version contains supplementary material available at 10.1186/s13244-023-01405-w.

## Background

Although the incidence of cervical cancer (CC) in the developed countries has decreased for the past two decades, it remains the fourth most common cancer and the leading cause of cancer-related death worldwide in women [1]. Radical hysterectomy and pelvic lymphadenectomy are the standard treatment options for patients with early-stage cervical cancer(ECC) [2]. However, studies demonstrated that there is less than 30% ECC with pelvic lymph node metastasis (LNM), which indicates over 70% patients with ECC were overtreated and suffered from unnecessary complications of lymphadenectomy [3, 4]. It is necessary to identify LNM accurately for patients with ECC in order to avoid unnecessary lymphadenectomy. Sentinel lymph node (SLN) biopsy has been suggested to decrease the need of pelvic lymphadenectomy for patients with ECC [5, 6]. The accuracy of SLN biopsy can be improved by several types of ancillary methods, including immunohistochemistry, polymerase chain reaction and serial sectioning [5–8]. However, both lymphadenectomy and SLN biopsy are invasive modalities; therefore, noninvasive and accurate prediction of LNM is very important for patients with ECC.

Magnetic resonance imaging (MRI) is the mainstay image modality for the staging of CC, especially the apparent diffusion coefficient (ADC) values derived from diffusion weighted imaging (DWI) are increasing applied to characterize the tumor microenvironment and microstructure of CC for LNM diagnosis [9]. However, traditional MRI mainly assesses the sizes of lymph nodes and is limited in its diagnosis sensitivity, which may lead to inappropriate treatment decisions [10]. With the emergence of radiomics, many attempts had been reported using different combinations of MRI in the preoperative prediction of LNM for patients with CC, such as T2 weighted imaging sequences (T2WI) [11–13], T2WI combined with dynamic contrast-enhanced MRI (DCE) [14–16], multiple-parameters MRI [17–19], ADC [20], as well as T2WI combined with ADC for locally advanced CC [21]. Although the sensitivity of MRI in the prediction of LNM has been improved with radiomics, the overall performance of these studies ranged from poor to moderate, particularly, radiomics features extracted from lymph nodes rather than primary tumor in some studies may affect the stability of radiomics features resulting from the relatively small volume of lymph nodes [22].

The purpose of this study is to investigate the accuracy and sensitivity of intra- and peritumoral radiomics features in T2WI, DWI sequences for preoperative LNM prediction with ECC patients and develop a flexible nomogram with clinical factors for potential clinical utilities.

## Materials and methods

### Patients

**Fig. 1:**
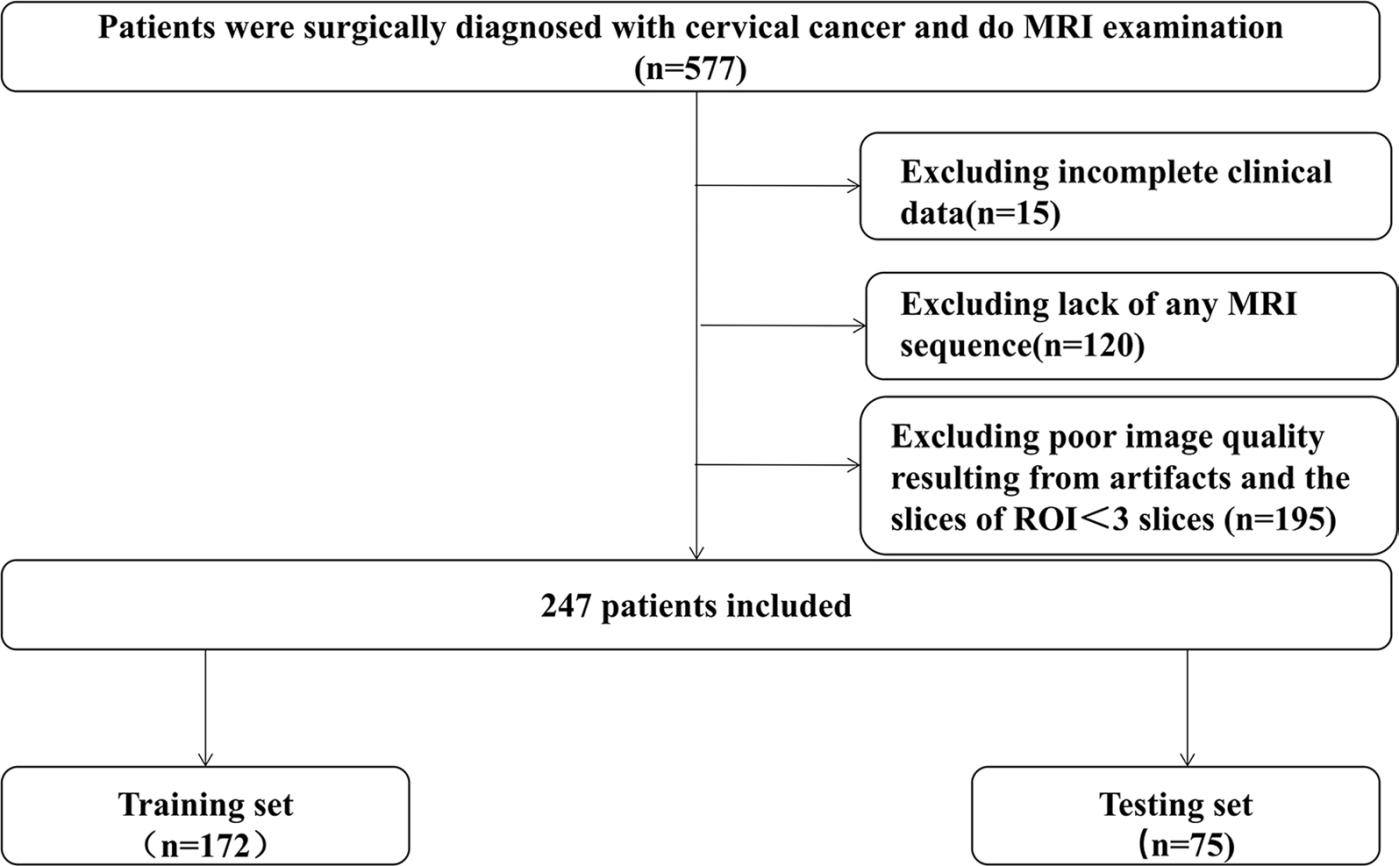
The flowchart for patients recruitment in this study

Patients with ECC from January 2008 and December 2018 were retrospectively reviewed and analyzed through searching electronic medical records in the authors’ hospital. The inclusion criteria were: (1) patients underwent radical hysterectomy and systematic pelvic lymph node dissection; (2) with histologically confirmed ECC according to the International Federation of Gynecology and Obstetrics (FIGO) stage; (3) without any treatment before surgery; (4) with detailed clinical and pathological characteristic; (5) standard MRI examination less than 2 weeks before hysterectomy. The exclusion criteria were: (1) incomplete clinical data; (2) lack of sufficient MRI sequences: T2WI with fat suppression or DWI; (3) poor image quality (including artifacts and the slices of ROI < 3 slices) [14]. The patients’ selection flowchart is shown in Fig. 1. The Ethics Committee in Clinical Research (ECCR) of the authors’ hospital approved this study, which was conducted in accordance with the Declaration of Helsinki (ECCR no. 2019059), and waived the need of written informed consent with confirmation of patient data confidentiality due to nature of the retrospective study.

### MRI acquisition and segmentation

MR images of sagittal T2WI with fat suppression and transverse DWI were acquired through a 3.0 T scanner (PHILIPS, ACHIEVA) using a 16-channel phased array body coil. T2WI was acquired applying parameters of TR/TE = 3000/90 ms, FOV = 220 × 220 mm, matrix = 336 × 336, thickness = 4 mm and gap = 1 mm with fast spin echo sequence. DWI was acquired applying parameters of TR/TE = 3000/55 ms, FOV = 430 × 334 mm, matrix = 224 × 224, thickness = 4 mm, and gap = 1 mm in transverse orientation including the entire female pelvis with a single-shot echo planar imaging (EPI) sequence using a b value of 0 and 1000 s/mm^2^. All images were stored in a picture archiving and communication system (PACS).

**Fig. 2:**
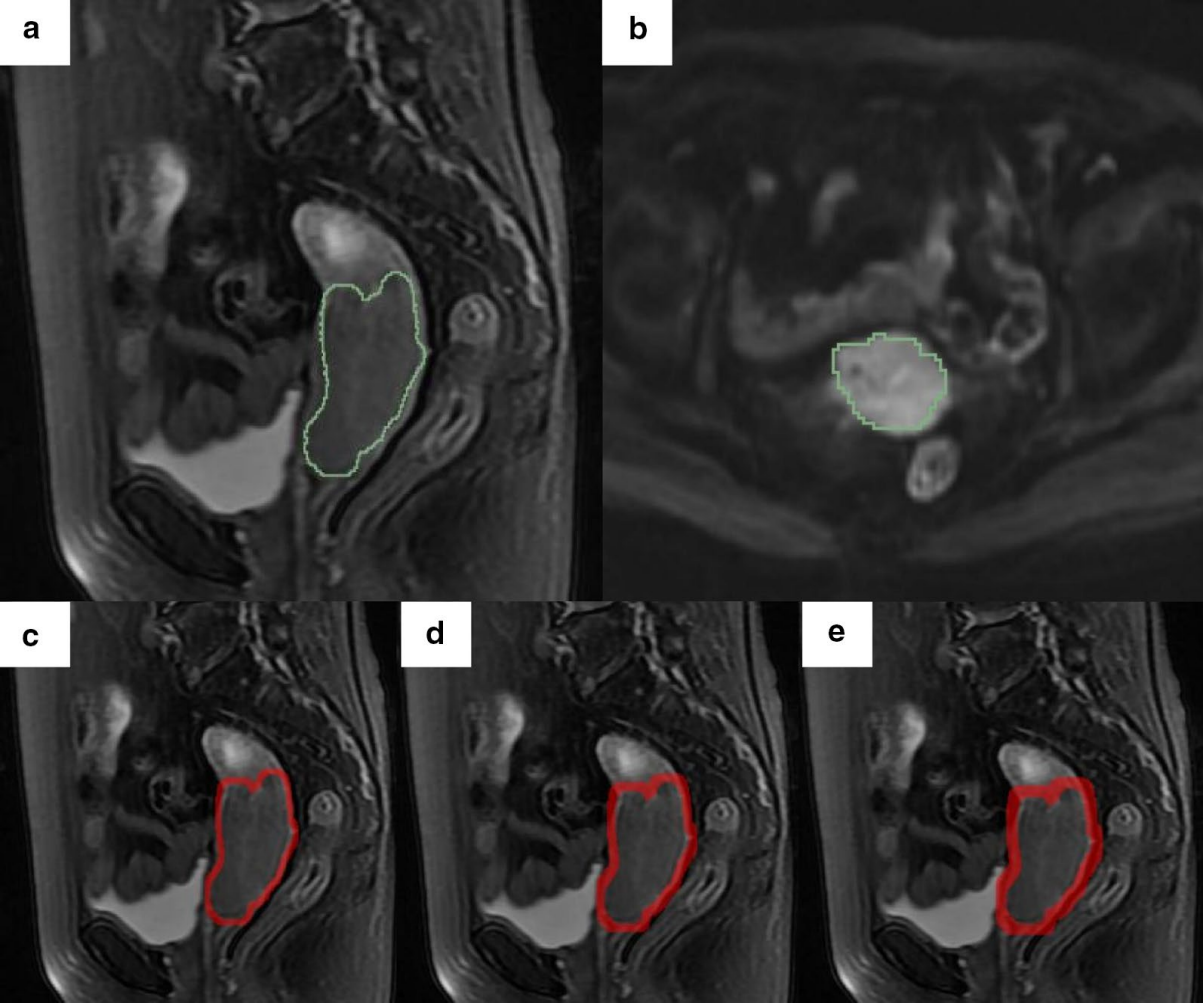
The intra- and peritumoral ROIs in this study. **a** is on sagittal T2WI; **b** is on DWI. The green line represents the boundary of the tumor; **c**-**e** are the example (3, 5, 7 mm expansion dimension) of the dilated MRI with various radial dilation distances outside the original masks in the MRI. The red rings indicate the peritumoral regions

The regions of interest (ROIs) of tumor were manually delineated along the boundary of the tumor slice by slice in sagittal T2WI with fat suppression and transverse DWI by a radiologist with 7 years of experience via a 3D Slicer software (version 4.2.1, https://www.slicer.org). A mask of the area with tumor boundary was defined as the intra-tumoral region. The peritumoral ROIs were obtained with python (version 3.7.6) by uniform expansion of intra-tumoral region with a dimension of 3 mm, 5 mm, 7 mm, respectively [23, 24]. The tumor boundary to the outer expansion boundary was defined as peri-tumoral region. In order to ensure the accuracy of segmentation, in the process of image segmentation, a senior radiologist has already validated all uncertain segmentation. Typical ROIs of intra- and peritumoral regions in sagittal T2WI with fat suppression and transverse DWI are shown in Fig. 2.

### Radiomics feature extraction and selection

In preprocessing, intensity normalization was performed to transform arbitrary gray intensity values into a standardized intensity range in T2WI and DWI. Radiomics features of first-order, shape-based, texture, Laplacian of Gaussian and wavelet features were extracted from contoured intra- and peritumoral ROIs using Python package (Pyradiomics) according to Image Biomarker Standardization Initiative (IBSI) [25]. Mann–Whitney U tests were firstly used to select potentially informative features with a *p* < 0.05. Secondly, the least absolute shrinkage and selection operator (LASSO) method was applied to select the optimal features through tuning the coefficient λ and using ten-fold cross-validation to avoid over-fitting.

### Radiomics signature and nomogram

Radiomics signatures were constructed with selected optimal features and their nonzero coefficients from intra- and peritumoral ROIs, as well as their combinations for the prediction of LNM status. The performance of radiomics signatures was evaluated by receiver operating characteristic (ROC) curves and the value of area under curve (AUC). Univariate analysis was performed to select clinical factors that associated with LNM of ECC. A nomogram was constructed using multivariate logistic regression integrating clinical factors and radiomics signature for the preoperatively prediction of LNM of ECC. A C-index was calculated in the training and testing sets to assess the discrimination performance of the nomogram. Calibration curves were plotted to evaluate the calibration of the radiomics nomogram with 1000 bootstrap resamples, and the goodness of fit was assessed with the Hosmer–Lemeshow (H–L) test [26].

### Statistics analysis

Statistical analyses were performed in R software (version 3.6.0, http://www.Rproject.org). Key radiomics features selection and logistic regression modeling were done using the “glmnet” package. Nomogram construction and calibration plots were performed using “rms” package. The H–L test was performed using “Resource Selection” package. The ROC curves were calculated using Medcalc software. For continuous clinical variables, two sample t test was used to assess the equality of variances between positive and negative LNM groups. For categorical variables, fisher’s exact test and Chi-square test were used to test the difference between groups. For all tests, *p* < 0.05 was considered as statistically significant.

## Results

A total of 247 patients with ECC were enrolled in this study with a mean age of 54.1 ± 10.01 (range, 28–77), as shown in Fig. 1 for the patients enrollment. There were 79 patients with LNM and 168 of non-LNM, respectively. Patients were randomly divided into training and testing sets by a ratio of 7 to 3 with 172 patients (mean age ± SD, 53.91 ± 9.88; range,28–77) and 75 patients (mean age ± SD, 54.54 ± 10.35; range, 30–75) allocated to the training and testing sets, respectively. There was no significant difference in LNM status between the training and testing sets (31.9% vs. 32%, *p* = 0.92). Detailed clinicopathologic characteristics of patients are shown in Table 1.

**Table 1:** Characteristics of the enrolled patients in the training and testing sets

Characteristic	Training set (*n* = 172)		*p*	Testing set (*n* = 75)		*p*
	LNM (*n* = 55)	Non-LNM (*n* = 117)		LNM (*n* = 24)	Non-LNM (*n* = 51)	
Age (mean ± SD)			0.083			0.64
Mean	51.80	54.91		55.54	54.04	
SD	11.68	8.79		10.77	10.23	
FIGO stage			0.69			0.64
I	25 (45.45%)	57 (48.72%)		9 (37.5%)	22 (43.14%)	
II	30 (54.55%)	60 (51.28%)		15 (62.5%)	29 (56.86%)	
Pathological type			0.95			0.086
SCC	46 (83.64%)	99 (84.61%)		20 (83.34%)	43 (84.31%)	
AC	5 (9.09%)	8 (6.84%)		2 (8.33%)	8 (15.69%)	
Others	4 (7.27%)	10 (8.55%)		2 (8.33%)		
MTD			0.016			0.009
≤ 4 cm	36 (65.45%)	96 (82.05%)		15 (62.50%)	45 (88.24%)	
> 4 cm	19 (34.55%)	21 (17.95%)		9 (37.50%)	6 (11.76%)	
LVSI			< 0.001			0.01
Negative	27 (49.09%)	91 (77.78%)		13 (54.17%)	42 (82.35%)	
Positive	28 (50.91%)	26 (22.22%)		11 (45.83%)	9 (17.65%)	

LNM: lymph node metastasis; Non-LNM: without lymph node metastasis; SD: standard deviation; FIGO: International Federation of Gynecology and Obstetrics; MTD: maximum tumor diameter; SCC: Squamous cell carcinoma; AC: Adenocarcinoma; LVSI: lymph-vascular space invasion

A total of 1014 radiomics features were extracted from the intra-, peritumoral ROIs with 3 mm, 5 mm and 7 mm expansion in T2WI and DWI, respectively. After Mann–Whitney U test, there were 676, 442, 409, 462 and 645, 387, 298, 285 radiomics features that were selected with intra-, peritumoral ROIs with 3 mm, 5 mm, and 7 mm expansion in T2WI and DWI. By applying LASSO method, a total of 17, 12, 12, 12 and 9, 15, 5, 11 optimal radiomics features were remained, respectively. The details are shown in Additional file 1: Fig. S1. Additional file 1: Table S1 shows the selected radiomics features and their corresponding nonzero coefficients. Radiomics score (Radscore) was calculated by multiplying the features with their corresponding nonzero coefficients and then adding them together. The Radscore based on intra-, peritumoral ROIs with 3 mm, 5 mm, and 7 mm expansion in T2WI and DWI for each patient is shown in Additional file 1: Fig. S2.

**Fig. 3:**
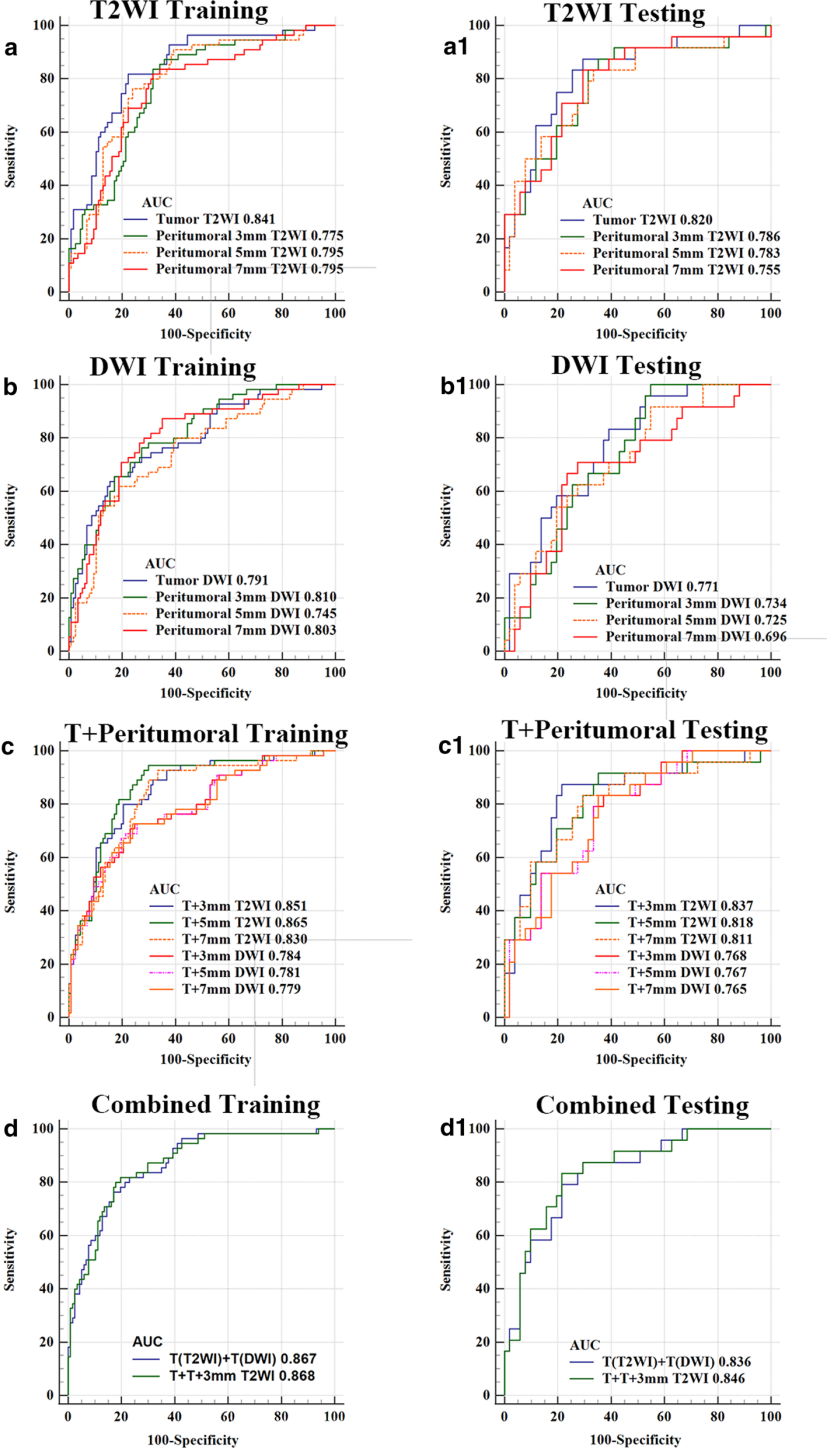
ROC curves of intra- and peritumoral regions with (3, 5, 7 mm) expansion dimension, intra-tumoral region plus peritumoral regions with (3, 5, 7 mm) expansion dimension on T2WI and DWI, combining intra-tumor regions in T2WI and DWI, combined radiomics signature from tumors in the T2WI, DWI and peritumoral 3 mm expansion in T2WI in the (**a-d**) training and (**a1-d1**) testing sets, respectively

The AUCs of radiomics signature with features from tumors in T2WI and DWI were 0.841 (0.779–0.904) vs. 0.791 (0.718–0.865) and 0.820 (0.715–0.926) vs. 0.771 (0.664–0.879) in the training and testing sets, respectively. The best performance of radiomics signature with features extracted from peritumoral ROIs was peritumoral 3 mm expansion in T2WI and DWI with an AUC of 0.786 (0.669–0.903) and 0.734 (0.623–0.846) in the testing sets, respectively. Combining radiomics with features from tumor and peritumoral expansion achieved a best AUC of 0.837 (0.733–0.940) and 0.768 (0.659–0.877) in the testing set for tumor plus peritumoral 3 mm expansion in the T2WI and DWI, respectively. Combined radiomics signature from tumors in the T2WI, DWI and peritumoral 3 mm expansion in T2WI achieved an AUC of 0.868 (0.809 -0.915) and 0.846 (0.753–0.940) in the training and testing sets, respectively. Detailed performance of these models is presented in Table 2 and Fig. 3.

**Table 2:** Performance of radiomics signature with features extracted from tumor and peritumoral regions of interest

Models	Training sets				Testing sets			
	AUC (95% CI)	ACC	SEN	SPE	AUC (95% CI)	ACC	SEN	SPE
T2WI								
Tumor	0.841 (0.779–0.904)	0.791	0.778	0.818	0.820 (0.715–0.926)	0.760	0.706	0.875
Peritumoral 3 mm	0.775 (0.702–0.847)	0.733	0.684	0.836	0.786 (0.669–0.903)	0.720	0.647	0.875
Peritumoral 5 mm	0.795 (0.724–0.866)	0.762	0.761	0.764	0.783 (0.664–0.903)	0.720	0.783	0.833
Peritumoral 7 mm	0.795 (0.727–0.862)	0.762	0.798	0.680	0.755 (0.624–0.886)	0.787	0.898	0.577
DWI								
Tumor	0.791 (0.718–0.865)	0.773	0.829	0.650	0.771 (0.664–0.879)	0.680	0.608	0.833
Peritumoral 3 mm	0.810 (0.743–0.876)	0.738	0.727	0.764	0.734 (0.623–0.846)	0.613	0.451	0.958
Peritumoral 5 mm	0.745 (0.666–0.825)	0.750	0.812	0.618	0.725 (0.605–0.844)	0.600	0.451	0.917
Peritumoral 7 mm	0.803 (0.733–0.873)	0.720	0.65	0.873	0.696 (0.567–0.826)	0.720	0.725	0.708
T + 3 mm T2WI	0.851 (0.790–0.912)	0.797	0.795	0.800	0.837 (0.733–0.940)	0.813	0.784	0.875
T + 5 mm T2WI	0.865 (0.807–0.923)	0.780	0.701	0.945	0.818 (0.710–0.926)	0.733	0.647	0.917
T + 7 mm T2WI	0.830 (0.765–0.896)	0.750	0.667	0.927	0.811 (0.702–0.921)	0.747	0.706	0.833
T + 3 mm DWI	0.784 (0.710–0.860)	0.744	0.752	0.727	0.768 (0.659–0.877)	0.693	0.627	0.833
T + 5 mm DWI	0.781 (0.705–0.856)	0.721	0.786	0.691	0.767 (0.657–0.877)	0.707	0.647	0.833
T + 7 mm DWI	0.779 (0.703–0.855)	0.750	0.761	0.727	0.765 (0.655–0.874)	0.707	0.647	0.833
T (T2WI) + T(DWI)	0.867 (0.810–0.924)	0.802	0.829	0.746	0.836 (0.741–0.931)	0.760	0.706	0.875
T + T + 3 mm T2WI	0.868 (0.809–0.915)	0.800	0.803	0.817	0.846 (0.753–0.940)	0.808	0.784	0.833

**Fig. 4:**
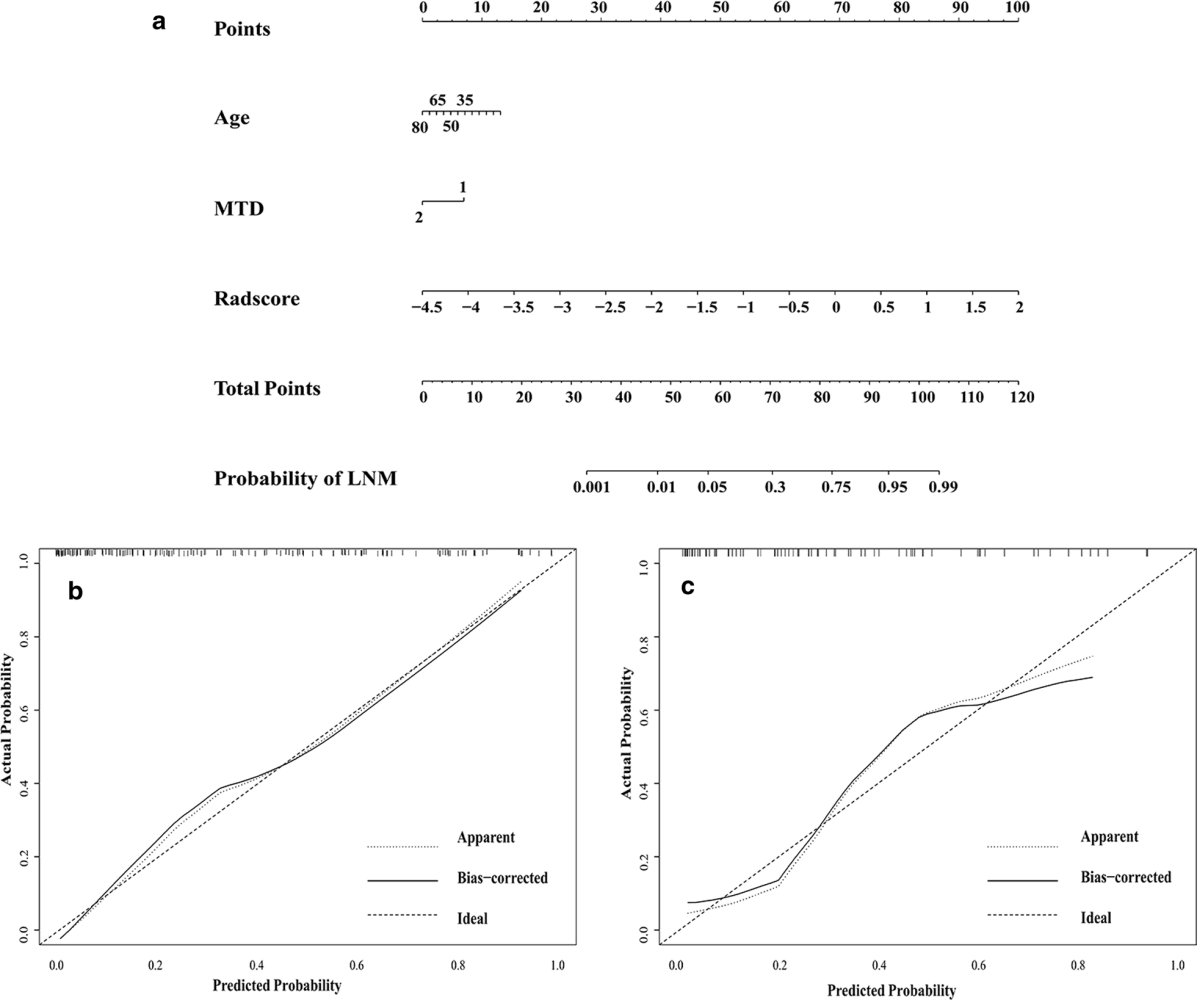
Nomogram was developed by integrating the clinical factors (age and MTD) and combined radiomics signature in the training set (**a**); Calibration curve of the radiomics nomogram for predicting LNM in the (**b**) training set (*p* = 0.55) and in the (**c**) testing set (*p* = 0.14)

T2WI: T2 weighted imaging; DWI: diffusion-weighted imaging; AUC: area under curer; ACC: accuracy; SEN: sensitivity; SPE: specificity; T + xmm: tumor plus peritumoral x mm expansion;

As shown in Table 3, the univariate analysis of clinical factors that associated with LNM of ECC, age and MTD were selected and combined with radiomics signature to develop a nomogram using multivariate logistic regression to further improve the prediction on the LNM status for ECC. The lymph-vascular space invasion (LVSI) was not included as it can be obtained only postoperatively. As shown in Fig. 4a, the nomogram achieved a C-index of 0.884 (95% CI,0.831–0.937) in the prediction of LNM for patients with ECC. The calibration curves are shown in Fig. 4b, c with no statistical significance observed in the H–L test (*p* = 0.55 and *p* = 0.14).

**Table 3:** Univariate analysis of clinical factors that were associated with lymph node metastasis

Variables	Odds ratios	95% confident interval	*p*
Age	0.968	0.936–1.001	0.056
FIGO stage	1.140	0.599–2.168	0.69
Pathological type	0.996	0.608–1.631	0.99
MTD	2.413	1.164–5.003	0.018
LVSI	3.630	1.829–7.201	< 0.001

FIGO: International Federation of Gynaecology and Obstetrics; MTD: maximum tumor diameter; LVSI: lymph-vascular space invasion

## Discussion

In this study, radiomics features extracted from the tumor regions in T2WI and DWI achieved an AUC of 0.820 and 0.771, respectively, in the prediction of LNM for patients with ECC. Combined features from tumor regions with additional features extracted from peritumoral 3 mm expansion in T2WI improved the AUC to 0.846 in the LNM prediction. A C-index of 0.884 was achieved with a nomogram integrating combined radiomics signature with clinical factors in the prediction of LNM for patients with ECC.

LNM is one of the most important prognostic factors for patients with ECC with a reported 5-year survival rate of 55% vs. 90% for patients with vs. without LNM [27]. LNM is also important evidence for treatment decision with chemoradiation rather than surgery as their first choice to avoid possible serious complications [28]. In this study, 79 (31.9%) of the enrolled 247 patients were confirmed with LNM, which is a bit higher than reported statistics of less than 30% of ECC with LNM [28]. This may be due to that 134 out of 247 ECC patients (54.24%) enrolled in this study were of FIGO stage II or patients selection bias due to a large number of patients excluded because of image quality or incomplete MRI sequences. Therefore, accurately identifying the LNM preoperatively is of critical clinical importance in the management of patients with ECC.

T2WI, contrasted-enhanced TIWI and DWI have long been applied for the diagnosis of LNM with a relatively low sensitivity of 38%-56% reported [29, 30]. In this study, the sensitivity for LNM prediction was improved from 0.647 to 0.898, and 0.451 to 0.725 for radiomics models with features from T2WI and DWI in the testing, respectively. Models with combined features from tumor and peritumoral regions did not improve the sensitivity in this study. This is inconsistent with the study of Wu et al. [21] in which the sensitivity was increased from 43% to 85.7% for models with features extracted from tumor or peritumoral regions alone compared with models with combined tumor and peritumoral regions features in T2WI. For features extracted from DWI, the combined model showed a worse sensitivity compared with models with features extracted from tumor or peritumoral regions. This may be due to the differences in the definition of peritumoral regions between two studies. Automatic expansion with 3–7 mm margin was applied in this study, instead of a manual segmentation of peritumoral regions in the study of Wu et al. [21].

In this study, the AUCs of radiomics model with features extracted from tumors in the T2WI and DWI were 0.841, 0.791 and 0.820, 0.771 in the training and testing sets, respectively. This is higher than reported AUC of 0.763, 0.829 and 0.699, 0.613 in the training and validation sets with T2WI and ADC, respectively, in the study of Hou et al. [17] for the prediction of LNM for ECC. Combined T2WI and DWI model in this study achieved an AUC of 0.836 in the testing sets which is close to the reported AUC of 0.833 with combined T2WI, ADC and contrast-enhanced T1WI in the study of Hou et al. [31]. However, Yu et al. [20] demonstrated that radiomics features extracted from ADC maps alone were able to achieve an AUC of 0.870 in the predicting of LNM for early-stage cervical squamous cell carcinoma. This indicated that radiomics features extracted from DWI and ADC maps revealed different messages although ADC maps were calculated from DWI and the selection of b-values may significantly affect texture analysis on DWI images [31].

The radiomics models with features extracted from peritumoral regions in T2WI and DWI also demonstrated reasonable an AUC from 0.755 to 0.786, and from 0.696 to 0.734 in the testing sets, respectively. This indicated that peritumoral regions may hold information regarding the LNM status as pointed out that tumor cells tend to migrate from the primary tumor to the peritumoral regions and lead to morphological changes in MRI [32]. However, the prediction performance did not improve with increasing peritumoral region and the peritumoral 3 mm in T2WI and DWI achieved the highest prediction performance, which may suggest that the increase in peritumoral region brings some irrelevant information; for example, the peritumoral 5 mm and 7 mm regions of some FIGO II stage patients may contain other tissues affecting the prediction performance. Combined radiomics models with tumor and peritumoral 3 mm regions further improved the performance in both T2WI and DWI in this study with a highest AUC of 0.868 and 0.846 achieved in the training and testing sets, respectively. This is consistent with the study of Shi et al. [23] in which both intra- and peritumoral regions of contrast-enhanced T1WI and T2WI were combined to achieve an AUC of 0.830 and 0.853.

Radiomics nomogram with multi-parametric MRI, such as T1WI, T2WI, contrast-enhanced, DWI, and ADC, has been reported to achieve a good performance for LNM prediction with a C-index of 0.882 in the primary cohorts [18]. Similarly, the nomogram in this study achieved a C-index of 0.884 and calibration curve analysis further confirmed the clinical usefulness of our nomogram for the preoperative LNM prediction. Except for MRI based radiomics, ultrasound images [33], ^18^fluorodeoxyglucose positron emission tomography/computed tomography (PET/CT) had also been investigated for the preoperative prediction of LNM for ECC [34]. Nomogram with radiomics features from multiple imaging modalities needs further investigation. Recently, deep learning models had also been investigated to predict the LNM of ECC to avoid time consuming target delineations in radiomics, whose clinical application and integration with radiomics worth further investigation [19].

The limitations of this study include that it is a retrospective study in single center, fewer patients recruited and lacking of the external validation may lead to over-fitting of the models, larger sample size from multi-center is needed to improve the robust of the models; large number of patients excluded due to artifacts and incomplete MRI sequences may lead to bias in patients selection, and the scanning technique needs to be further improved and standardized; lacking of doing intraclass correlations coefficient analysis may affect the stability of feature selection and the robust of the model, and it will be improved in the following study; contrast-enhanced T1WI and ADC maps were not included in this work; automatic segmentation needs further investigation to improve the efficiency of radiomics study.

## Conclusions

Radiomics features extracted from both intra- and peritumoral regions in T2WI and DWI are feasible and promising for the preoperative prediction of LNM for patients with ECC.

## Supplementary Information

**Additional file 1**. Radiomic feature selection and the optimal features from different ROIs in T2WI and DWI; Radscores for each patient in the intra- and peritumoral regions with (3,5,7mm) expansion dimension on T2WI and DWI.

## Data Availability

The datasets used and/or analyzed during the current study are available from the corresponding author on reasonable request.

## Abbreviations

AC: Adenocarcinoma
ADC: Apparent diffusion coefficient
AUC: Area under the curve
CC: Cervical cancer
DCE: Dynamic contrast enhanced
DWI: Diffusion weighted imaging
ECC: Early-stage cervical cancer
ECCR: Ethics committee in clinical research
EPI: Echo planar imaging
FIGO: International federation of gynecology and obstetrics
H–L: Hosmer–Lemeshow
IBSI: Image biomarker standardization initiative
LASSO: Least absolute shrinkage and selection operator
LNM: Lymph node metastasis
LVSI: Lymph-vascular space invasion
MRI: Magnetic resonance imaging
MTD: Maximum tumor diameter
PACS: Picture archiving and communication system
PET/CT: Positron emission tomography/computed tomography
Radscore: Radiomics score
ROC: Receiver operating characteristic
ROIs: Regions of interest
SCC: Squamous cell carcinoma
SLN: Sentinel lymph node
T2WI: T2 weighted imaging
