# Supplementary material for: Intra- and peri-tumoral MRI radiomics features for preoperative lymph node metastasis prediction in early-stage cervical cancer
Source: Insights Imaging. 2023 Apr 15;14:65. doi: 10.1186/s13244-023-01405-w (PMC10105820; doi:10.1186/s13244-023-01405-w)
Supplement: Supplementary file 1 — Additional file 1. Radiomic feature selection and the optimal features from different ROIs in T2WI and DWI; Radscores for each patient in the intra- and peritumoral regions with (3,5,7mm) expansion dimension on T2WI and DWI. [file 13244_2023_1405_MOESM1_ESM.pdf]

ELECTRONIC SUPPLEMENTARY MATERIAL

Intra- and peri-tumoral MRI radiomics features for preoperative lymph node metastasis prediction in early-stage cervical cancer

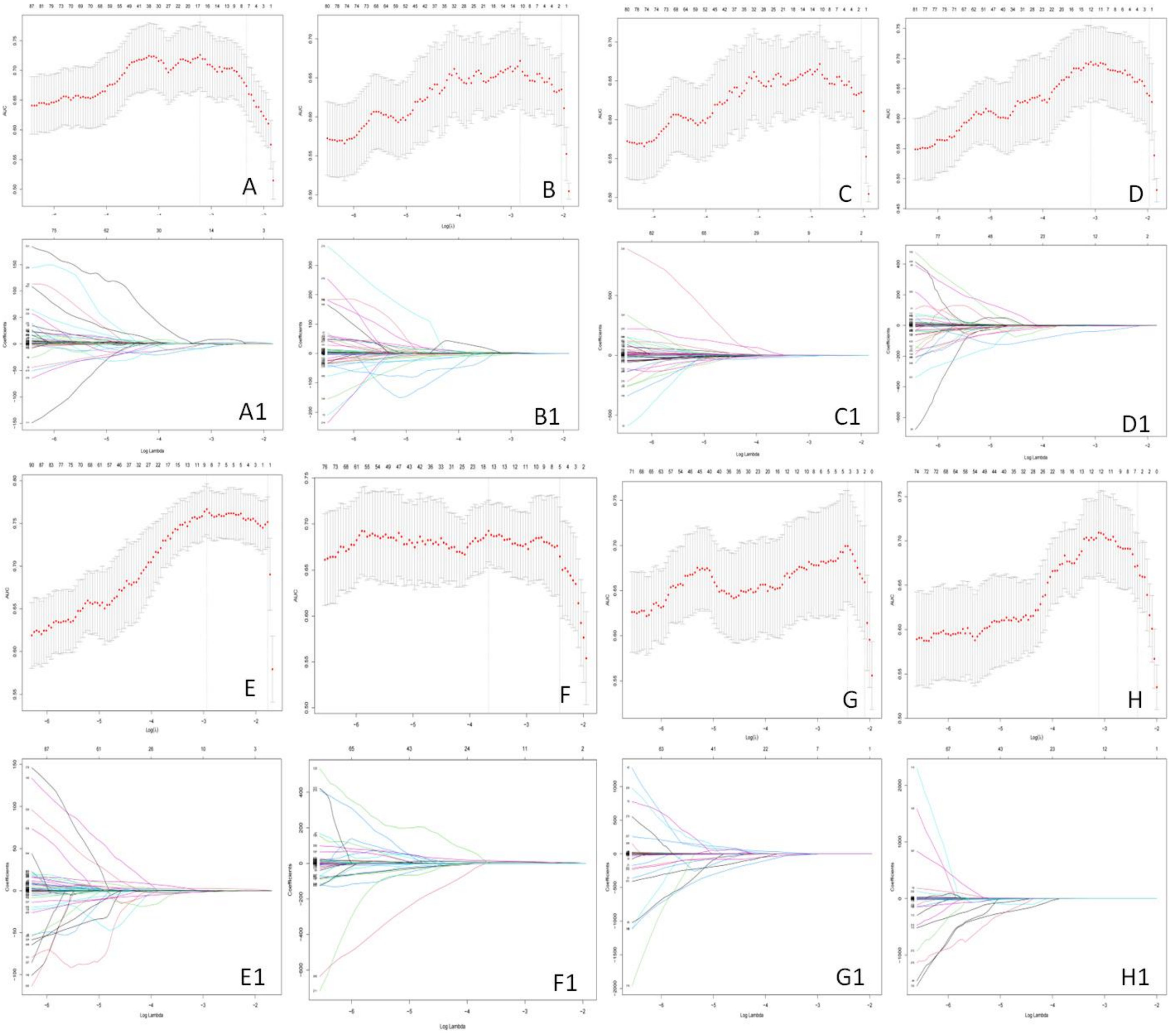

Figure S1. Radiomic feature selection using the least absolute shrinkage and selection operator (LASSO) regression method in T2WI. The images (A-H) show that tuning parameter lambda (λ) is chosen in the LASSO model using 10-fold cross-validation via minimum criteria. The area under the curve (AUC) was plotted versus log (λ). Dotted vertical lines were drawn at the minimum criteria and the 1 standard error of the minimum criteria (the 1-SE criteria). The images (A1-H1) show the LASSO coefficient profiles of the features. A coefficient profile plot was produced against the log (λ) sequence. (A-D, A1-D1) and (E-H, E1-H1) are for intra- and peritumoral regions with (3, 5, 7 mm) expansion dimension in T2WI and DWI respectively.

ELECTRONIC SUPPLEMENTARY MATERIAL

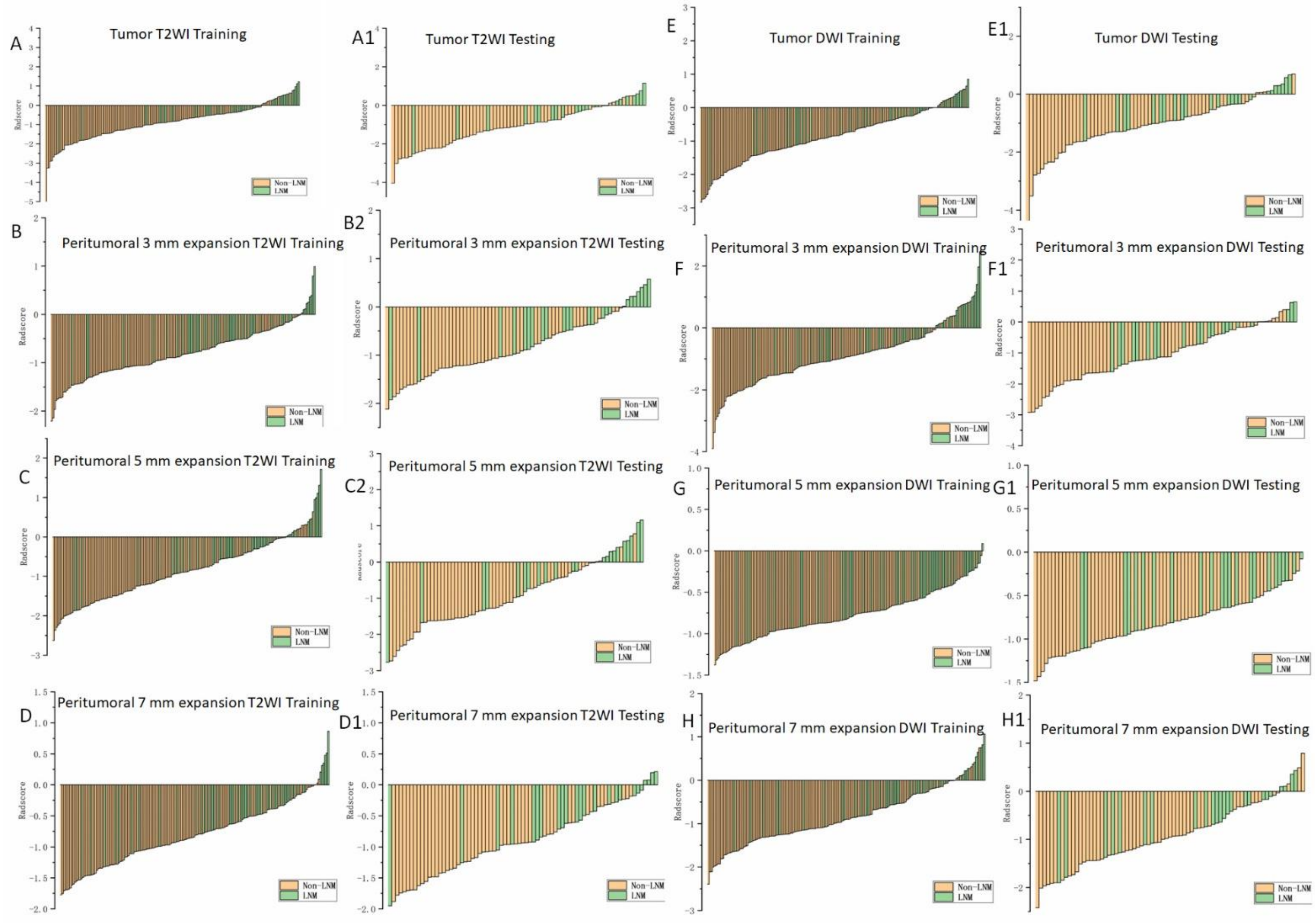

Figure S2. Radscores for each patient in the intra- and peritumoral regions with (3, 5, 7mm) expansion dimension on T2WI and DWI in the (A-H) training and (A1-H1) testing sets respectively. The green bars indicate the LNM patients. The orange bars indicate the patients without LNM.

ELECTRONIC SUPPLEMENTARY MATERIAL

Table S1. Selected optimal features from different ROIs in the T2WI and DWI.

Number	Feature	coefficient
**Tumor T2WI**		
1	original_shape_Elongation	1.3599797739
2	original_shape_Maximum2DDiameterColumn	0.0056583131
3	original_glcm_Correlation	0.6741388024
4	original_glszm_GrayLevelNonUniformityNormalized	-0.3415433719
5	log.sigma.3.0.mm.3D_glrlm_LongRunLowGrayLevelEmphasis	-0.5724790079
6	log.sigma.5.0.mm.3D_firstorder_90Percentile	0.0082949253
7	wavelet.LHL_firstorder_Mean	0.0056946492
8	wavelet.LHL_glcm_Correlation	1.0898060399
9	wavelet.LHH_firstorder_10Percentile	0.0077637980
10	wavelet.LHH_glrlm_LongRunLowGrayLevelEmphasis	-0.7711876720
11	wavelet.HLL_firstorder_Median	0.0043533472
12	wavelet.HLL_firstorder_Skewness	-0.0084754702
13	wavelet.HLH_glcm_Idmn	4.4075679451
14	wavelet.HLH_glrlm_LongRunLowGrayLevelEmphasis	-0.2596862423
15	wavelet.HHL_firstorder_Kurtosis	0.0194539717
16	wavelet.HHL_firstorder_Maximum	0.0008670239
17	wavelet.LLL_firstorder_Minimum	-0.0019256261
**Peritumoral 3mm expansion in T2WI**		
1	original_shape_LeastAxisLength	0.01397134
2	original_shape_Maximum2DDiameterColumn	0.002623524
3	original_shape_Sphericity	-0.5328199
4	original_firstorder_Minimum	-6.179416e-03
5	original_shape_SurfaceVolumeRatio	0.1372844
6	wavelet.LLH_firstorder_Median	8.966571e-03
7	wavelet.LHL_glcm_Idmn	1.467265
8	wavelet.HLH_gldm_LargeDependenceHighGrayLevelEmphasis	6.201872e-05
9	wavelet.HHL_ngtdm_Complexity	1.172836e-07
10	wavelet.LLL_glcm_Correlation	0.9559049
11	wavelet.HLH_gldm_LargeDependenceHighGrayLevelEmphasis	6.201872e-05
12	wavelet.HHL_gldm_LargeDependenceHighGrayLevelEmphasis	1.988707e-05
**Peritumoral 5 mm expansion in T2WI**		
1	original_shape_Elongation	0.6194996
2	original_shape_Maximum2DDiameterColumn	2.911261e-03
3	original_shape_Sphericity	-1.256590
4	original_firstorder_Minimum	-8.243181e-03
5	log.sigma.5.0.mm.3D_glszm_LargeAreaLowGrayLevelEmphasis	-2.382767
6	wavelet.LHL_glcm_Imc2	-1.636070

7	wavelet.LHH_ngtdm_Strength	-1.156373e-02
8	wavelet.HLH_glszm_SmallAreaLowGrayLevelEmphasis	-12.47921
9	wavelet.HLH_gldm_LargeDependenceHighGrayLevelEmphasis	3.132259e-05
10	wavelet.HHL_gldm_LargeDependenceHighGrayLevelEmphasis	5.857471e-05
11	wavelet.HHH_glcm_ClusterShade	1.388996e-04
12	wavelet.LLL_glcm_Correlation	2.306193

Peritumoral 7 mm expansion in T2WI

1	original_shape_Flatness	0.2.445876
2	original_shape_Sphericity	-0.625721
3	original_firstorder_Minimum	-3.444680e-03
4	log.sigma.5.0.mm.3D_glszm_SmallAreaLowGrayLevelEmphasis	-0.14735
5	wavelet.LHH_ngtdm_Contrast	-0.5436369
6	wavelet.HLH_glcm_JointEnergy	-0.080240
7	wavelet.HLH_ngtdm_Complexity	4.821825e-05
8	wavelet.HHL_glcm_DifferenceVariance	3.502376e-04
9	wavelet.HHL_gldm_LargeDependenceHighGrayLevelEmphasis	2.988182e-05
10	wavelet.HHH_glcm_Idn	2.932004
11	wavelet.LLL_firstorder_RobustMeanAbsoluteDeviation	4.036665e-04
12	wavelet.LLL_glcm_Correlation	0.6067658

Tumor in DWI

1	log.sigma.3.0.mm.3D_firstorder_90Percentile	0.004672143
2	log.sigma.3.0.mm.3D_firstorder_RootMeanSquared	0.001982266
3	log.sigma.5.0.mm.3D_firstorder_Maximum	0.001881352
4	wavelet.LLH_firstorder_Skewness	-0.100452399
5	wavelet.LLH_glcm_Correlation	0.378456413
6	wavelet.HLL_firstorder_Mean	0.002071771
7	wavelet.HLH_glszm_SmallAreaEmphasis	-1.310917850
8	wavelet.HHL_glcm_Correlation	-0.588285775
9	wavelet.LLL_glcm_Correlation	2.571251173

Peritumoral 3mm expansion in DWI

1	original_shape_MajorAxisLength	3.845579e-03
2	original_shape_MinorAxisLength	2.517041e-02
3	original_gldm_LargeDependenceHighGrayLevelEmphasis	3.357256e-05
4	log.sigma.3.0.mm.3D_firstorder_Skewness	7.292574e-01
5	log.sigma.3.0.mm.3D_glcm_Correlation	8.755779e-01
6	log.sigma.5.0.mm.3D_firstorder_Maximum	1.683162e-03
7	log.sigma.5.0.mm.3D_glcm_Correlation	0.5227037
8	log.sigma.5.0.mm.3D_glcm_Idn	7.541583
9	wavelet.LHL_glcm_MaximumProbability	9.910835
10	wavelet.LHH_glszm_ZoneVariance	4.010247e-04
11	wavelet.HLH_firstorder_Minimum	3.092103e-03
12	wavelet.HLH_glrlm_RunEntropy	-0.1392890

13	wavelet.HHL_firstorder_Mean	0.1047677
14	wavelet.HHH_firstorder_Skewness	-0.055775
15	wavelet.LLL_glcm_Idn	15.87004
**Peritumoral 5 mm expansion in DWI**		
1	DWI_peritumor_5mmoriginal_shape_MinorAxisLength	1.857630e-02
2	original_gldm_LargeDependenceHighGrayLevelEmphasis	2.924143e-06
3	log.sigma.3.0.mm.3D_glcm_Correlation	1.328039
4	wavelet.HLH_ngtdm_Busyness	5.196560e-02
5	wavelet.LLL_firstorder_Minimum	-8.632634e-04
**Peritumoral 7 mm expansion in DWI**		
1	original_shape_MinorAxisLength	0.0337557109
2	original_shape_Sphericity	-0.0554105771
3	log.sigma.3.0.mm.3D_glcm_Correlation	0.8487627726
4	log.sigma.5.0.mm.3D_firstorder_Maximum	0.0001759538
5	log.sigma.5.0.mm.3D_glcm_Correlation	1.7677994366
6	wavelet.HLL_firstorder_InterquartileRange	0.0040446322
7	wavelet.HLL_firstorder_Skewness	-0.5093463515
8	wavelet.HLH_ngtdm_Busyness	0.9099190073
9	wavelet.HHL_firstorder_Skewness	0.5165364854
10	wavelet.HHH_firstorder_Skewness	-0.7447985342
11	wavelet.LLL_firstorder_Minimum	-0.0017443127
